# Inconsistent Methods Used to Set Airway Pressure Release Ventilation in Acute Respiratory Distress Syndrome: A Systematic Review and Meta-Regression Analysis

**DOI:** 10.3390/jcm13092690

**Published:** 2024-05-03

**Authors:** Mark R. Lutz, Jacob Charlamb, Joshua R. Kenna, Abigail Smith, Stephen J. Glatt, Joaquin D. Araos, Penny L. Andrews, Nader M. Habashi, Gary F. Nieman, Auyon J. Ghosh

**Affiliations:** 1 Department of Surgery, SUNY Upstate Medical University, Syracuse, NY 13210, USAcharlaja@upstate.edu (J.C.); kennajo@upstate.edu (J.R.K.); 2 Health Sciences Library, SUNY Upstate Medical University, Syracuse, NY 13210, USA; smithab@upstate.edu; 3 Department of Psychiatry and Behavioral Sciences, SUNY Upstate Medical University, Syracuse, NY 13210, USA; 4 Department of Neuroscience and Physiology, SUNY Upstate Medical University, Syracuse, NY 13210, USA; 5 Department of Public Health and Preventive Medicine, SUNY Upstate Medical University, Syracuse, NY 13210, USA; 6 Department of Clinical Sciences, College of Veterinary Medicine, Cornell University, Ithaca, NY 14853, USA; jda246@cornell.edu; 7 Department of Critical Care, R Adams Cowley Shock Trauma Center, Baltimore, MD 21201, USA; 8 Division of Pulmonary, Critical Care, and Sleep Medicine, SUNY Upstate Medical University, Syracuse, NY 13210, USA; ghosha@upstate.edu

**Keywords:** ARDS, VILI, APRV

## Abstract

Airway pressure release ventilation (APRV) is a protective mechanical ventilation mode for patients with acute respiratory distress syndrome (ARDS) that theoretically may reduce ventilator-induced lung injury (VILI) and ARDS-related mortality. However, there is no standard method to set and adjust the APRV mode shown to be optimal. Therefore, we performed a meta-regression analysis to evaluate how the four individual APRV settings impacted the outcome in these patients. **Methods:** Studies investigating the use of the APRV mode for ARDS patients were searched from electronic databases. We tested individual settings, including (1) high airway pressure (P_High_); (2) low airway pressure (P_Low_); (3) time at high airway pressure (T_High_); and (4) time at low pressure (T_Low_) for association with PaO_2_/FiO_2_ ratio and ICU length of stay. **Results:** There was no significant difference in PaO_2_/FiO_2_ ratio between the groups in any of the four settings (P_High_ difference −12.0 [95% CI −100.4, 86.4]; P_Low_ difference 54.3 [95% CI −52.6, 161.1]; T_Low_ difference −27.19 [95% CI −127.0, 72.6]; T_High_ difference −51.4 [95% CI −170.3, 67.5]). There was high heterogeneity across all parameters (P_hHgh_ I^2^ = 99.46%, P_Low_ I^2^ = 99.16%, T_Low_ I^2^ = 99.31%, T_High_ I^2^ = 99.29%). **Conclusions:** None of the four individual APRV settings independently were associated with differences in outcome. A holistic approach, analyzing all settings in combination, may improve APRV efficacy since it is known that small differences in ventilator settings can significantly alter mortality. Future clinical trials should set and adjust APRV based on the best current scientific evidence available.

## 1. Introduction

Acute respiratory distress syndrome (ARDS) is a heterogeneous disorder that arises from a variety of pulmonary and extrapulmonary insults and is uniformly associated with high mortality [1,2]. Current treatment is supportive, including mechanical ventilation, prone positioning, and extracorporeal membrane oxygenation [3]. Despite several advances in the role of mechanical ventilation in the mitigation of lung injury, incorrectly adjusted ventilator settings can lead to unintended ventilator-induced lung injury (VILI), which has been shown to increase mortality in ARDS [4].

Repetitive alveolar collapse and expansion (RACE) is the primary cause of atelectrauma, which, along with volutrauma, comprise the two main mechanical mechanisms of VILI [4,5]. Low tidal volume ventilation (LTVV) has been shown to reduce mortality in ARDS, ostensibly by limiting volutrauma in the remaining normal tissue. However, even after the application of LTVV, mortality from ARDS remains unacceptably high [6,7,8]. Therefore, there is a need for novel protective modes of ventilation that can ameliorate both volutrauma and atelectrauma [NO_PRINTED_FORM].

Airway pressure release ventilation (APRV) is a form of mechanical ventilation that has been shown to have a superior physiologic profile compared to conventional LTVV in pre-clinical studies [9]. Specifically, a time-controlled adaptive ventilation protocol for setting the APRV mode, which is personalized based on changes in respiratory system compliance (C_RS_), has several important plausible mechanisms to reduce the propagation of lung injury [10]. These mechanisms include: (i) a brief expiratory time personalized to lung pathophysiology (C_RS_) that stabilizes lung tissue via pressure and time, and (ii) the extended inspiratory time that leads to the recruitment of small volumes of lung tissue with each breath. In the first mechanism, the lung does not have time to fully depressurize, generating a time-controlled PEEP. In the second mechanism, lung tissue is gradually “ratcheted” open over time, while the brief expiratory time prevents the newly opened lung tissue from re-collapsing. These elements of the physiologic rationale for APRV stem, in part, from the literature supporting the use of higher vs. lower positive end-expiratory pressure (PEEP) strategies [11,12]. Accordingly, recent practice guidelines, supported by the American Thoracic Society (ATS), suggest using higher PEEP in moderate to severe ARDS [13]. Given the inverse ratio nature of APRV, PEEP tends to be lower, but the resulting increase in mean airway pressure approximates the goal of higher PEEP in normal ratio ventilation strategies. However, clinical trials have demonstrated mixed results, with a recent meta-analysis comparing APRV, using multiple protocols to set the mode, to LTVV, finding that APRV was associated with overall improved outcomes, albeit with relatively poor study quality [14].

One of the factors that contribute to the variability in APRV vs. LTVV clinical trials may be the lack of an optimal or even standardized protocol for initiating and adjusting APRV settings on a mechanical ventilator. We therefore sought to examine the effect of the four main APRV settings on outcomes in ARDS. The objectives of our study were to (i) identify if there were studies that used similar methods to set APRV and, if so, (ii) whether there is a method of setting APRV that was associated with an improvement in ARDS-related outcomes. We analyzed the four settings used to adjust APRV, including the highest level of pressure applied to the respiratory system (P_high_), the lowest level of pressure applied to the respiratory system (P_low_), and the time spent at each pressure setting (T_high_ and T_low_, respectively). We hypothesized that personalizing the four settings to lung pathophysiology (C_RS_) would be associated with reduced mortality and length of stay and improved oxygenation.

## 2. Methods

### 2.1. Search Strategy

This systematic review and meta-regression is reported according to the Preferred Reporting for Items for Systematic Reviews and Meta-analyses (PRISMA) guidelines. A health sciences librarian with experience in systematic reviews and literature searching developed the search strategies in consultation with the research team. Systematic search queries related to ARDS and APRV using a combination of keywords and controlled vocabulary (where available) were conducted in PubMed, Embase, Scopus, Web of Science Core Collection, and CENTRAL. The included databases are standard among medical systematic reviews and meta-analyses, and we chose not to include other, specialty-specific databases that are not representative of critical care to avoid additional publication biases. All databases and registers were searched from inception to 12 October 2023. There were no search restrictions on language, publication status, or outcomes. The details of all the search strategies, including specific terms and Boolean operators, can be found in Supplementary Materials File S1. The search identified 2131 records. Duplicates were removed using EndNote 20 (Endnote, Clarivate, available at www.endnote.com, accessd on 1 January 2023). The remaining 1755 records were uploaded into Covidence (Covidence, Veritas Health Innovation, Melbourne, Australia; available at ww.covidence.org) for title and abstract screening.

### 2.2. Study Inclusion Criteria and Outcomes Measured

We included all experimental studies that used APRV as a mode of ventilation within their study. All patients were adults (age ≥18 years) who underwent mechanical ventilation. We excluded case reports, literature reviews, and conference proceedings as they generally lacked the data specificity necessary for our intended analyses. There were no search restrictions on language, publication status, or outcomes. All studies were conducted on humans.

We chose to categorize ventilator settings into groups based on how each setting impacts lung physiology and their alignment with the preponderance of the basic science literature that has been shown to be lung-protective [10,15]. Specifically, we compared P_high_ set by plateau pressure vs. P_high_ not set by plateau pressure, P_low_ set to 0 vs. P_low_ not set to 0, T_low_ set to 50–75% of peak expiratory flow rate vs. T_low_ not set to 50–75% of peak expiratory flow rate, and T_high_ set based on PaCO_2_ vs. T_high_ set arbitrarily.

The primary outcomes of this study were mortality (percentage of patients surviving in the APRV group), P/F ratio (PaO_2_/FiO_2_), number of ventilator-free days, static lung compliance, and intensive care unit length of stay (LOS).

### 2.3. Data Extraction and Quality Assessment

All procedures were independently reviewed by three authors (JC, JK, ML) in accordance with the prespecified inclusion criteria. The general information extracted included study and subject characteristics (age, gender, etiology, study design, aim of study, inclusion and exclusion criteria, method of recruitment, ventilator mode, population size, and population characteristics), specific APRV settings, including the time at high pressure, the time at low pressure, the high pressure, and the low pressure (T_high_, T_low_, P_high_, P_low_), and outcome results. Data were recorded as either percentage or mean and standard deviation. The Cochrane Risk of Bias tool was used for the quality assessments. Any disagreements regarding data collection, data extraction, and quality assessment were resolved by consensus.

### 2.4. Statistical Analysis

Data extracted from the individual studies are presented as published (e.g., means and standard deviations) where available; when standard deviation (SD) was unavailable, we estimated the standard deviation (SD) from the range using a previously published method [16]. Between-study heterogeneity was assessed using the Cochran Q-test and I-squared test. We performed mixed-effects meta-regression to assess the association of APRV setting strategy and clinical outcomes of interest, using the maximum likelihood (ML) estimator for mixed-effect modeling. We chose to perform a meta-regression analysis as opposed to a traditional meta-analysis given the limitations of the meta-analysis framework. Specifically, a meta-analysis does not allow for a comparison of outcomes within a single arm (i.e., P_low_ set to 0 vs. not set to 0) as opposed to a comparison of outcomes between an intervention and a control (i.e., P_low_ set to 0 vs. LTVV). To maximize statistical power, we compared strategies in individual ventilator setting parameters (i.e., P_high_, P_low_, T_low_, T_high_) instead of groups of settings, because, given there is no standardized protocol and a limited number of studies available, there were several combinations of ventilator settings presented as APRV in individual studies. We similarly chose not to perform sub-group analyses due to the small number of studies across parameter combinations, which could limit the validity and interpretability of any tests of interaction between sub-groups. We defined statistical significance as a 2-sided alpha < 0.5. Statistical analysis was performed using the *metafor* R package in R (version 4.3.2, The R Foundation for Statistical Computation, Vienna, Austria).

## 3. Results

### 3.1. Study Selection and Characteristics

**Figure 1:**
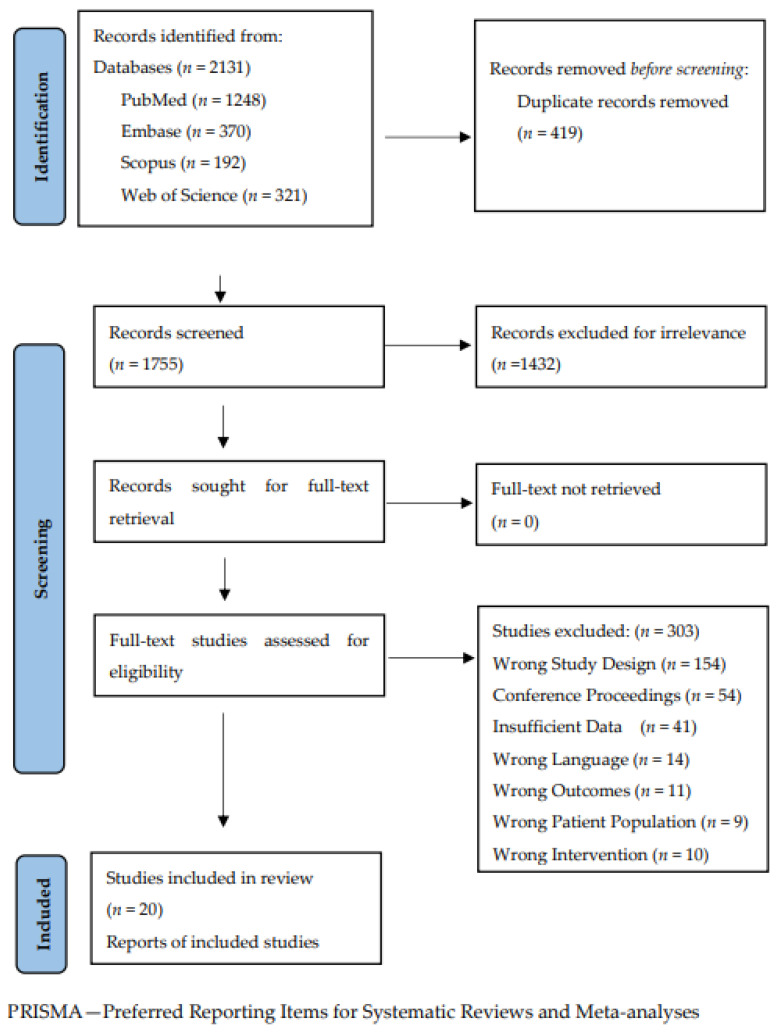
PRISMA flowchart.

We identified 2,131 records from a systematic search of the included databases (PubMed *n* = 1248, Embase *n* = 370, Scopus *n* = 192, Web of Science *n* = 321, CENTRAL *n* = 43). In total, 419 duplicative records were excluded. After screening 1755 titles and abstracts, 1432 records were excluded for irrelevance. After full-text assessment, 20 studies were included in our data extraction and subsequent analysis (Figure 1). The majority of studies excluded at full-text assessment (*n* = 303) were for incorrect study design (i.e., the study was not a RCT or cohort study), incorrect publication type (i.e., conference proceeding, and/or incomplete data). The remaining excluded studies were for languages other than English, incorrect outcomes (i.e., did not include any of the outcomes of interest), incorrect patient population or pediatric population, incorrect intervention, incorrect clinical setting, or repeat study.

We included 9 cohort studies, 10 randomized controlled trials, and one cross-over study (Table 1) [17,18,19,20,21,22,23,24,25,26,27,28,29,30,31,32,33,34,35,36]. The average number of participants from randomized controlled trials was 37 while the average number of participants from cohort studies was 22. The included studies were published between 1994 and 2022. Six of the included studies were conducted in the United States. The other studies were performed in Australia, China, Egypt, Finland, Germany, India, Japan, Mexico, and Turkey. The sample sizes ranged from 6 to 71, for a total of 538 study participants.

### 3.2. Bias and Study Quality Assessment

Table 2 documents the quality assessment of the included studies as determined through the Cochrane Risk of Bias tool. While most of the studies employed adequate random sequence generation and allocation concealment, the overwhelming majority of studies lacked blinding of both participants and personnel as well as an outcome assessment. We found that there was a very heterogeneous assessment of outcomes across the included studies. Most studies had complete outcome data for PaO_2_/FiO_2_ (P/F) ratio and intensive care unit (ICU) length of stay (LOS). However, the majority of studies did not include complete data for mortality, ventilator-free days, and static lung compliance. Therefore, we were only able to perform meta-regression analyses for the P/F ratio and ICU LOS. There was limited evidence of selective reporting, and we detected a low risk of other bias.

### 3.3. Meta-Regression Analyses

**Figure 2:**
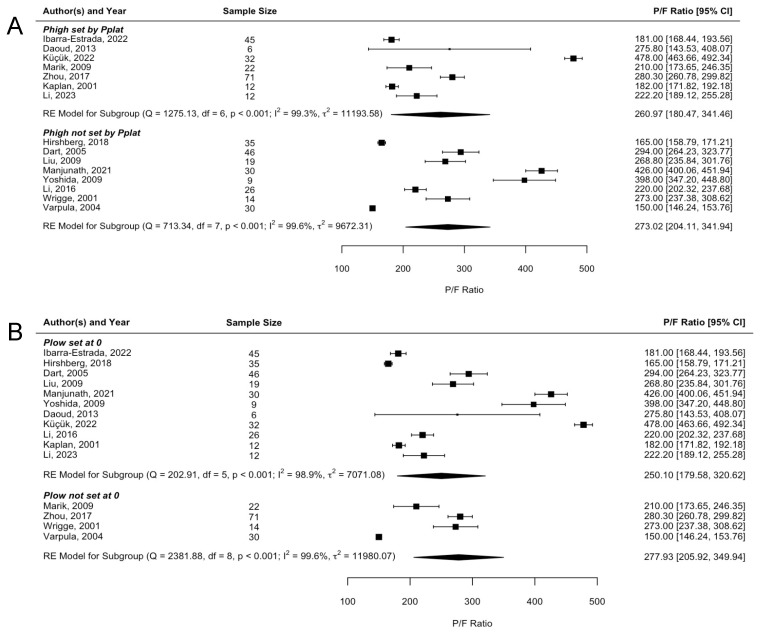

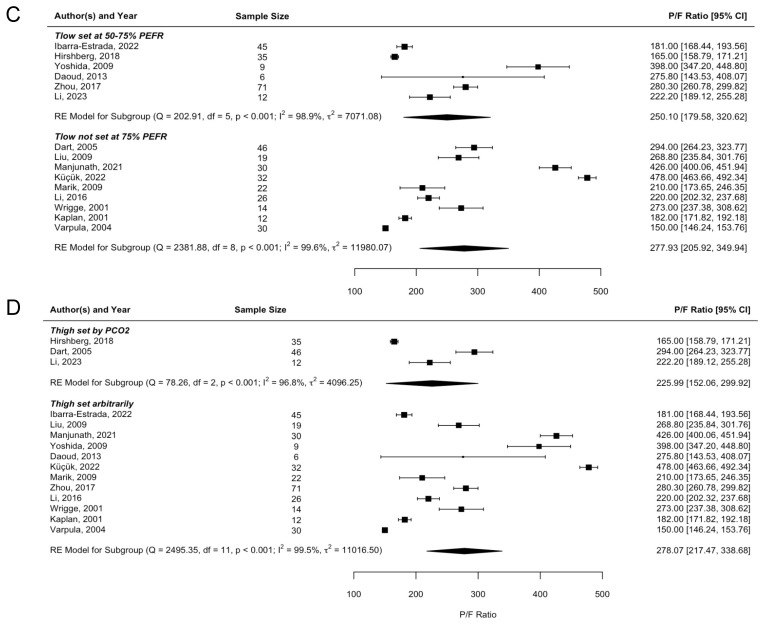
P/F ratio forest plots. Forest plots of P/F ratio analysis by APRV setting. (**A**) P_high_ forest plot. (**B**) P_low_ forest plot. (**C**) T_low_ forest plot. (**D**) T_high_ forest plot [17,18,19,20,21,22,23,24,25,26,27,28,29,30,31,32,33,34,35,36].

We performed mixed model meta-regression analyses with each ventilator setting individually as the categorical independent variable and outcome measure of interest as the dependent variable with a study-level random intercept to account for between-study heterogeneity. In total, 15 studies had complete data (i.e., both mean and SD) for the P/F ratio. There was no significant difference in P/F ratio between groups defined on the basis of any of the four settings (P_high_ difference −12.0 [95% CI −100.4, 86.39]; P_low_ difference 54.3 [95% CI −52.6, 161.1]; T_low_ difference −27.19 [95% CI −127.0, 72.6]; T_high_ difference −51.4 [95% CI −170.3, 67.5]). The forest plots are shown in Figure 2. There was high heterogeneity across all parameters (P_high_ I^2^ = 99.46%, P_low_ I^2^ = 99.16%, T_low_ I^2^ = 99.31%, T_high_ I^2^ = 99.29%).

**Figure 3:**
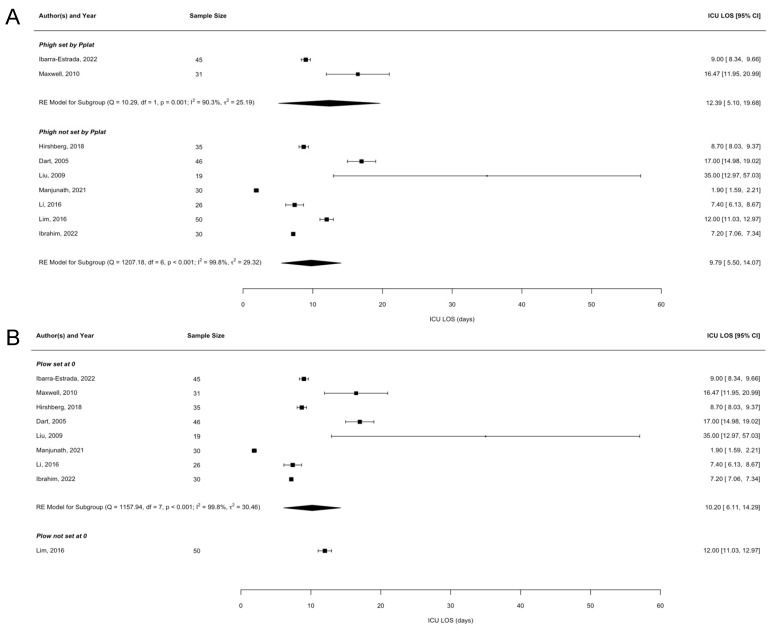

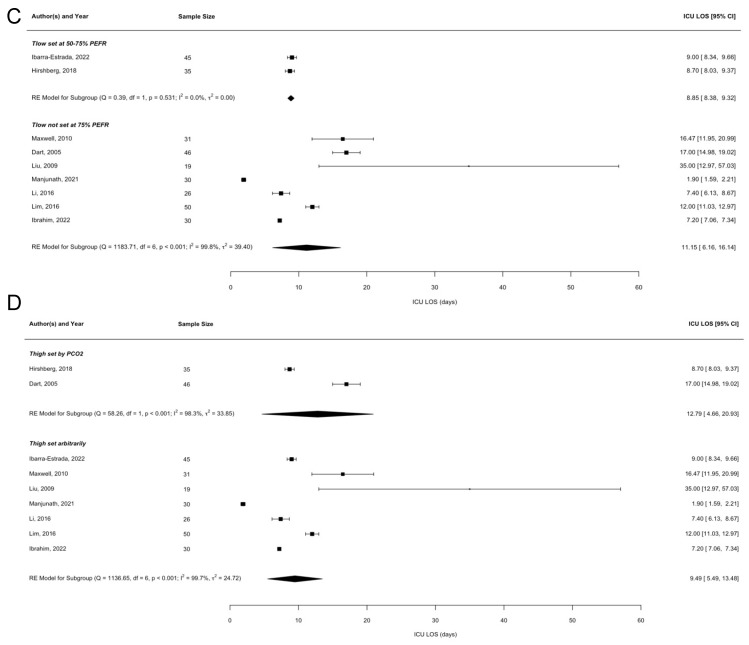
ICU LOS forest plots. Forest plots of ICU LOS analysis by APRV setting. (**A**) P_high_ forest plot. (**B**) P_low_ forest plot. (**C**) T_low_ forest plot. (**D**) T_high_ forest plot [17,18,19,20,21,22,23,24,25,26,27,28,29,30,31,32,33,34,35,36].

There were 9 studies with complete data for ICU LOS. Similarly, there was no significant difference in ICU LOS between the groups in any of the four settings (P_high_ difference 2.8 days [95% CI −4.8, 10.3], P_low_ difference −2.0 days [95% CI −11.7, 7.7], T_low_ difference −1.8 days [95% CI −9.4, 5.7], T_high_ difference 3.5 days [95% CI −3.5, 10.4]). The forest plots are shown in Figure 3. As with the P/F ratio analysis, there was high heterogeneity across all four ventilator setting parameters (P_high_ I^2^ = 99.63%, P_low_ I^2^ = 99.67%, T_low_ I^2^ = 99.63%, T_high_ I^2^ = 99.59%).

## 4. Discussion

In our systematic review and meta-regression analysis, we found no difference in oxygenation or ICU LOS between strategies in setting individual APRV-related ventilator parameters. In addition, we also demonstrate significant variability in the setting and adjustment of APRV in patients with ARDS. Taken together, these findings suggest that the most effective combination of APRV settings has not been established, and the design of existing trials comparing APRV to LTVV may be suboptimal.

The variability in APRV settings among the included studies was one of the most striking findings of our study. Among the 20 studies included in our analyses, 11 of the 16 possible combinations of the 4 ventilator settings were represented, with only 2 combinations represented by more than 2 studies each. Given the inconsistency in APRV protocols used across the studies and the limited number of studies in each combination, comparisons of APRV to LTVV are limited by study heterogeneity, and comparisons of APRV protocols are limited by the low number of studies with matching ventilator setting configurations. We attempted to address some of the between-study variability by pursuing a meta-regression analysis of single APRV arms instead of the traditional meta-analysis comparing APRV to LTVV. Given the relatively even distribution of studies within each parameter, aside from T_low_, we were similarly able to employ meta-regression analysis to circumvent some of these issues.

We categorized APRV settings based on extensive physiologic data from studying the four APRV setting categories in animal models of ARDS. Although ARDS pathophysiology is highly complex, the dynamic change in alveolar mechanics predisposes the lung to a secondary VILI. In this setting, the lung becomes time- and pressure-dependent, such that it takes more time to open lung tissue and less time to re-collapse at any given airway pressure [37]. Thus, a longer inspiratory time (T_High_) and a very brief expiratory time (T_Low_) may rapidly stabilize and then gradually recruit collapsed lung tissue, eliminating both atelectrauma and volutrauma.

Arguably, the most important APRV setting is the T_Low_. If the T_Low_ is set to be sufficiently brief, it will not allow sufficient time for the alveoli to collapse, even with very rapid collapse time constants. Preventing alveolar collapse during expiration has two important lung-protective benefits: (1) progressive lung collapse moving the lung into the “VILI Vortex” would be prevented, and (2) atelectrauma, a primary VILI mechanism, would be minimized [4,38]. The slope of the expiratory flow curve (Slope_EF_) has been shown to be a measure of C_RS_ and can be used to personalize the T_Low_ based on changes in lung pathophysiology [39]. Importantly, the expiratory flow curve is a breath-by-breath assessment of C_RS_ only when the P_low_ is set to 0 cmH_2_O. We have previously demonstrated that if the expiratory flow is terminated (T_EF_) at 75% of the peak expiratory flow (P_EF_) (P_EF_ L/min x 75% = T_EF_ L/min), alveolar collapse and dynamic heterogeneity is prevented, whereas increasing the T_Low_ (P_EF_ x 10%, 25%, or 50%) does not. We have further shown that using this method to set T_Low_ is highly lung-protective both in a clinically relevant large animal model and in clinical case series [40,41,42]. Despite the volume of data supporting its use, only one study included in our present analysis used P_EF_ 75% to set the T_Low_ [36].

In addition, we have previously demonstrated that, when using P_EF_ 75% to set T_Low_, alveoli are stabilized even when P_Low_ was set at 0 cmH_2_O [43]. Interestingly, the majority of studies included in the present analysis set P_Low_ to 0 cmH_2_O (Table 1). Using this method, T_Low_ is sufficiently brief such that the lung does not have time to fully depressurize. Therefore, alveolar stability is maintained by a combination of time and pressure. While P_Low_ is set at 0 cmH_2_O, the end-expiratory pressure remains approximately half of the P_High_ value [44]. On the other hand, P_Low_ set above 0 cmH_2_O has two negative effects: (i) the added resistance slows the expiratory flow, and the Slope_EF_ is no longer an accurate assessment of C_RS_, and (ii) the reduced rate of expiration may cause an increase in PaCO_2_.

APRV can be adjusted to increase PaCO_2_ removal using two basic methods that increase minute ventilation. The T_Low_ could be increased to augment tidal volume (V_T_), but from a physiologic perspective, this is problematic. It is well known that a large V_T_ can cause VILI, and lengthening the T_Low_ can cause alveolar instability (atelectrauma) and alveolar duct overdistension (volutrauma) [8,43,45]. Alternatively, reducing the length of the T_High_ to increase respiratory rate can increase PaCO_2_ removal. While decreasing T_High_ is likely more lung protective than increasing T_Low_, which would increase V_T_ and compromise alveolar stability, the shorter inspiratory time may slow progressive lung recruitment. Only three of the studies analyzed in this meta-analysis changed T_High_ to eliminate PaCO_2_ (Table 1).

One of the main limiting factors of existing clinical trials comparing APRV to LTVV has been sample size. Assuming an effect size comparable to the ARMA trial (approximately 10% reduction in mortality), α = 0.05, and 80% power, the estimated sample size needed would be over 700 individuals. Among the studies included in our analysis, the largest sample size was 71 individuals, with a total of 538 individuals across all included studies, which only makes up just above 60% of the estimated sample size of an adequately powered clinical trial. Any future trial will thus need to address these considerations to study APRV in the clinical setting appropriately.

The primary limitation of our analysis was the substantial heterogeneity in the included studies. The I^2^ for all the analyses was over 99%, suggesting that almost all the observed variation was due to between-study, as opposed to within-study, differences. As mentioned above, at least some and perhaps a large proportion of the heterogeneity is clearly due to the multiple combinations of parameters across the studies. However, rather than showing that all APRV protocols are the same, our study demonstrates that the optimal strategy has not yet been demonstrated. There are also likely many other unknown sources of variability due to the diverse etiologies and clinical presentations of ARDS. For example, the study by Ibarra-Estrada et al. included only individuals with ARDS secondary to the novel coronavirus disease 2019 (COVID-19). While there are several clinical and biologic similarities between COVID-19 ARDS and non-COVID-19 ARDS, there are notable pathophysiologic differences, including the severity of endothelial injury, microangiopathy, and thrombosis [46]. In addition, there is a growing body of literature delineating two molecular phenotypes that have distinct biological profiles and mortality trajectories. Post hoc analyses have not demonstrated the interaction between randomized treatment and phenotype, but future studies that are designed with these heterogeneous groups in mind are necessary to better understand the role of APRV and other management strategies in ARDS [47].

Our results further highlight heterogeneity in clinical trial design as an important barrier to scientific advancement in the practice of critical care. In particular, the lack of standardization in protocols for mechanical ventilation limits the generalizability of any given clinical trial and the comparability of clinical trials in meta-analysis. For example, despite a consistent signal toward reduced mortality and improved oxygenation with higher PEEP, the authors of the recent ATS practice guideline offered only a conditional recommendation for the use of higher PEEP due to heterogeneity noted in the meta-analyses [13]. Future trials designed to evaluate APRV as a ventilation strategy for ARDS should include a clear justification for the method of setting each individual parameter, rather than comparing ventilator modes in name only.

Despite the number of criticisms of the existing APRV trials, we acknowledge that the ideal clinical trial design is complex. There may be several viable approaches, but a multi-arm parallel-group design is likely the most familiar and straightforward method for studying the multiple parameters required for APRV [48]. In this scenario, each arm would consist of a given combination of settings for the duration of the study period. Alternatively, a stepped wedge with or without cluster randomization could achieve similar results.

The principal source of bias for the included studies was the lack of blinding for participants and personnel, which was unfortunately unavoidable due to the nature of the intervention. In addition, several of the outcomes of interest, including, notably, mortality, had missing data, precluding inclusion in our analysis. However, there was sufficient data to analyze both the P/F ratio and ICU LOS, an important physiologic outcome and an important patient-centered outcome, respectively.

In conclusion, we found no differences in outcomes in individual APRV parameter strategies. Specifically, 68% of the included studies set the expiratory time (T_low_) arbitrarily and without scientific rationale. Setting the expiratory time to a specific physiologic parameter, such as C_RS_, may be the most important of the four settings since, if sufficiently short, it may eliminate recruitment/derecruitment-induced atelectrama. Only one study set expiratory time to 75% of P_EF_; thus, we could not further assess this method in sub-group analysis. While our analysis had several strengths, including the inclusion of a wide breadth of studies and meta-regression study design, the main limitation was the profound heterogeneity between included studies. With mortality related to ARDS remaining unacceptably high, further investigation into novel ventilation strategies is imminently necessary. Understanding the physiologic impact of each setting, both individually and in combination, is critical to optimizing the lung-protective impact of APRV. Our findings suggest that future studies are needed to establish the optimal combination of APRV settings to improve ARDS-related patient outcomes.

## Figures and Tables

**Table 1:** Characteristics from included studies.

Study	Design	Sample Size of APRV Group	T_low_ (%)	P_low_ Set to 0	T_high_ Adjusted for PCO_2_	P_high_ Set Based on P_plat_	Outcomes
Momtaz 2022 [36]	RCT	-	75	No	No	Yes	1
Li 2023 [17]	Cohort study	12	50–75	Yes	Yes	Yes	1,2
Varpula 2004 [18]	RCT	30	Arbitrary	No	No	No	1, 2, 3
Kaplan 2001 [19]	Cohort study	12	Arbitrary	-	No	-	2
Ibrahim 2022 [20]	RCT	30	Arbitrary	Yes	No	No	1, 4, 5
Wrigge 2001 [21]	Cohort study	14	Arbitrary	No	No	No	2
Lim 2016 [22]	Cohort study	50	Arbitrary	No	No	No	1, 2, 5
Li 2016 [23]	RCT	26	Arbitrary	Yes	No	No	1, 2, 3, 4, 5
Zhou 2017 [24]	RCT	71	50–75	No	Yes	Yes	1, 2, 3, 5
Marik 2009 [25]	Cohort study	22	Arbitrary	No	No	Yes	1, 2
Kucuk 2022 [26]	RCT	32	Arbitrary	Yes	No	Yes	1, 2, 3, 5
Daoud 2013 [27]	Cohort study	6	50–75	Yes	No	Yes	2
Yoshida 2009 [28]	Cohort study	9	50–75	Yes	No	No	2
Manjunath 2021 [29]	RCT	30	Arbitrary	Yes	No	No	2, 4, 5
Sydow 1994 [30]	Cross-over study	18	Arbitrary	No	No	No	1
Liu 2009 [31]	Cohort study	19	Arbitrary	Yes	No	No	1, 2, 5
Dart 2005 [32]	Cohort study	46	Arbitrary	Yes	Yes	No	1, 2, 3
Hirshberg 2018 [34]	RCT	35	50–75	Yes	Yes	No	1, 2, 3, 5
Maxwell 2010 [33]	Cohort study	31	Arbitrary	Yes	No	Yes	1, 5
Ibarra-Estrada 2022 [35]	RCT	45	50–75	Yes	No	Yes	1, 2, 3, 5

Outcomes: 1—mortality (percentage of patients surviving in the APRV group); 2—P/F ratio (PaO_2_/FiO_2_); 3—number of ventilator-free days; 4—static lung compliance; 5—intensive care unit length of stay.

**Table 2:** Quality assessment of included studies.

Study ID	Sequence Generation (Selection Bias)	Allocation Concealment (Selection Bias)	Blinding of Participants and Personnel (Performance Bias)	Blinding of Outcome Assessment (Detection Bias)	Incomplete Outcome Data (Attrition Bias)	Selective Reporting (Reporting Bias)	Other Sources of Bias
Momtaz 2022 [36]	Low	Low	Low	Low	Low	Low	Low
Li 2023 [17]	Low	Unsure	Low	Low	Low	Low	Low
Varpula 2004 [18]	Low	Low	High	Unsure	Low	Low	Low
Kaplan 2001 [19]	Unsure	High	High	High	High	Unsure	Low
Ibrahim 2022 [20]	Low	Low	High	High	Low	Low	Low
Wrigge 2001 [21]	Unsure	Unsure	High	High	High	Unsure	Low
Lim 2016 [22]	Low	Low	Unsure	Unsure	Low	High	Low
Li 2016 [23]	Low	Low	Unsure	Unsure	Unsure	Low	Low
Zhou 2017 [24]	Low	Low	High	Unsure	Low	Low	Low
Marik 2009 [25]	Unsure	High	High	High	High	High	Low
Küçük 2022 [26]	Low	Low	High	High	Low	Low	Low
Daoud 2013 [27]	High	High	Low	High	Low	Low	Low
Yoshida 2009 [28]	High	High	High	High	High	Unsure	Low
Manjunath 2021 [29]	Unsure	Low	High	High	High	Low	Low
Sydow 1994 [30]	Low	Unsure	High	Unsure	Low	Unsure	Low
Liu 2009 [31]	Low	High	Unsure	Unsure	Low	Low	Low
Dart 2005 [32]	Unsure	Low	Low	Low	Low	Low	Low
Hirshberg 2018 [34]	Low	Low	Low	High	Low	Unsure	Low
Maxwell 2010 [33]	Low	Low	High	Unsure	Low	Unsure	Low
Ibarra-Estrada 2022 [35]	Low	Low	High	High	Low	Low	Low

## Data Availability

The original data presented in the study are openly available in PubMed, Embase, Scopus, Web of Science Core Collection, and CENTRAL.

## Supplementary Materials

The following supporting information can be downloaded at: https://www.mdpi.com/article/10.3390/jcm13092690/s1, File S1: Search Strategy Details
