# Supplementary material for: Inconsistent Methods Used to Set Airway Pressure Release Ventilation in Acute Respiratory Distress Syndrome: A Systematic Review and Meta-Regression Analysis
Source: J Clin Med. 2024 May 3;13(9):2690. doi: 10.3390/jcm13092690 (PMC11084500; doi:10.3390/jcm13092690)
Supplement: Supplementary file 1 [file jcm-13-02690-s001.zip › jcm-2897055-supplementary.pdf]

Supplementary Materials File S1

Search Strategy Details

PubMed

("Respiratory Distress Syndrome"[MeSH Terms] OR "Acute Lung Injury"[MeSH Terms] OR "acute respiratory distress"[Title/Abstract] OR "adult respiratory distress"[Title/Abstract] OR "shock lung*"[Title/Abstract] OR "acute lung injur*"[Title/Abstract] OR "ALI"[Title/Abstract]) AND ("Continuous Positive Airway Pressure"[Mesh] OR "Airway Pressure Release Ventilation"[Title/Abstract] OR "air pressure release ventilation"[Title/Abstract] OR APRV[Title/Abstract] OR "pressure high"[Title/Abstract] OR "pressure low"[Title/Abstract] OR "P High"[Title/Abstract] OR "PHigh"[Title/Abstract] OR "P Low"[Title/Abstract] OR "PLow"[Title/Abstract] OR "time high"[Title/Abstract] OR "time low"[Title/Abstract] OR "Thigh"[Title/Abstract] OR "T High"[Title/Abstract] OR "Tlow"[Title/Abstract] OR "T low"[Title/Abstract])

Embase

#9 #8 NOT [medline/lim]

#8 #4 AND #7

#7 #5 OR #6

#6 'airway pressure release ventilation':ti,ab OR 'air pressure release ventilation':ti,ab OR aprv:ti,ab OR 'pressure high':ti,ab OR 'pressure low':ti,ab OR 'p high':ti,ab OR 'phigh':ti,ab OR 'p low':ti,ab OR 'plow':ti,ab OR 'time high':ti,ab OR 'time low':ti,ab OR 'thigh':ti,ab OR 't high':ti,ab OR 'tlow':ti,ab OR 't low':ti,ab

#5 'airway pressure release ventilation'/exp

#4 #1 OR #2 OR #3

#3 'acute respiratory distress':ti,ab OR 'adult respiratory distress':ti,ab OR 'shock lung*':ti,ab OR 'acute lung injur*':ti,ab OR 'ali':ti,ab

#2 'acute lung injury'/exp

#1 'adult respiratory distress syndrome'/exp

CENTRAL

#1 MeSH descriptor: [Respiratory Distress Syndrome] explode all trees

#2 MeSH descriptor: [Acute Lung Injury] explode all trees
#3 “acute respiratory distress”:ti,ab OR “adult respiratory distress”:ti,ab OR shock NEXT lung*:ti,ab OR acute NEXT lung NEXT injur*:ti,ab OR “ali”:ti,ab

#4 #1 OR #2 OR #3

#5 MeSH descriptor: [Continuous Positive Airway Pressure] explode all trees

#6 “Airway Pressure Release Ventilation”:ti,ab OR “air pressure release ventilation”:ti,ab OR APRV:ti,ab OR “pressure high”:ti,ab OR “pressure low”:ti,ab OR “P High”:ti,ab OR “PHigh”:ti,ab OR “P Low”:ti,ab OR “PLow”:ti,ab OR “time high”:ti,ab OR “time low”:ti,ab OR “Thigh”:ti,ab OR “T High”:ti,ab OR “Tlow”:ti,ab OR “T low”:ti,ab

#7 #5 OR #6

#8 #4 AND #7

#9 "accession number" near pubmed

#10 "accession number" NEAR/2 Embase

#11 #8 NOT #9

#12 #11 NOT #10

Web of Science (SCI-Expanded; ESCI; CPCI-S; SSCI)

#3 #1 AND #2

#2 TS=(“airway pressure release ventilation” OR “air pressure release ventilation” OR aprv OR “pressure high” OR “pressure low” OR “p high” OR “phigh” OR “p low” OR “plow” OR “time high” OR “time low” OR “thigh” OR “t high” OR “tlow” OR “t low”)

#1 TS=(“acute respiratory distress” OR “adult respiratory distress” OR “shock lung*” OR “acute lung injur*” OR “ali”)

Scopus

(TITLE-ABS-KEY("acute respiratory distress" OR "adult respiratory distress" OR "shock lung*" OR "acute lung injur*" OR "ali")) AND (TITLE-ABS-KEY("airway pressure release ventilation" OR "air pressure release ventilation" OR aprv OR "pressure high" OR "pressure low" OR "p high" OR "phigh" OR "p low" OR "plow" OR "time high" OR "time low" OR "thigh" OR "t high" OR "tlow" OR "t low")) AND NOT INDEX(MEDLINE)
